# Effect of *Lactiplantibacillus plantarum* GKK1 Supplementation on Exercise-Induced Fatigue, Muscle Damage, and Recovery in Healthy Men: A Randomized, Double-Blind, Placebo-Controlled Trial

**DOI:** 10.3390/nu18172782

**Published:** 2026-08-25

**Authors:** Mon-Chien Lee, Chao-Yuan Chen, Ying-Ti Shih, You-Shan Tsai, Shih-Wei Lin, Yen-Lien Chen, Chin-Chu Chen, Chi-Chang Huang

**Affiliations:** 1 Graduate Institute of Sports Science, National Taiwan Sport University, Taoyuan City 333325, Taiwan; kurt0710@ntsu.edu.tw (M.-C.L.); row750118@ntsu.edu.tw (C.-Y.C.); 2 Center for General Education, Taipei Medical University, Taipei 110301, Taiwan; 3 Physical Education Office, National Taipei University of Business, Taipei 100025, Taiwan; 4 Office of Student Affairs, Hsin Sheng Junior College of Medical Care and Management, Taoyuan 32544, Taiwan; vian1976@hsc.edu.tw; 5 Biotech Research Institute, Grape King Bio Ltd., Taoyuan City 325002, Taiwan; youshan.tsai@grapeking.com.tw (Y.-S.T.); wei.lin@grapeking.com.tw (S.-W.L.); lan.chen@grapeking.com.tw (Y.-L.C.); 6 Institute of Food Science and Technology, National Taiwan University, Taipei City 106319, Taiwan; 7 Department of Bioscience Technology, Chung Yuan Christian University, Taoyuan City 320314, Taiwan; 8 Institute of BioPharmaceutical Science, National Sun Yat-Sen University, Kaohsiung City 804201, Taiwan

**Keywords:** *Lactiplantibacillus plantarum* GKK1, probiotics, exercise-induced muscle damage, fatigue, muscle recovery, oxidative stress, inflammation

## Abstract

Background: Exercise-induced fatigue (EIF) and exercise-induced muscle damage (EIMD) impair neuromuscular performance and delay post-exercise recovery. However, human evidence regarding the structural and functional recovery kinetics following *Lactiplantibacillus plantarum* GKK1 supplementation remains limited. Objective: This randomized, double-blind, placebo-controlled trial investigated whether 4 weeks of *L. plantarum* GKK1 supplementation improves functional recovery and modulates biochemical responses after an EIMD protocol in healthy men. Methods: Forty-eight healthy men with no regular exercise habits were randomly assigned to receive either placebo (*n* = 24) or L. plantarum GKK1 (two capsules daily, totaling 1.0 × 10^11^ CFU; *n* = 24) for 28 consecutive days. The trial was registered at ClinicalTrials.gov (NCT06893549). After supplementation, participants completed 100 maximal plyometric jumps. Countermovement jump (CMJ), isometric mid-thigh pull (IMTP), and Wingate anaerobic performance were assessed before EIMD and at 3, 24, and 48 h post-EIMD. Blood biomarkers of muscle damage, inflammation, oxidative stress, endocrine response, and sympathoadrenal activity were analyzed, and urinary 3-methylhistidine and creatinine were measured. Results: Compared with placebo, GKK1 supplementation was associated with smaller post-exercise decrements in the CMJ rate of force development, relative peak force, jump height, IMTP relative peak force and peak RFD, and Wingate anaerobic performance (*p* < 0.05). GKK1 also attenuated post-exercise increases in CK, myoglobin, hs-CRP, and TBARS, and was associated with more favorable testosterone, HGH, catecholamine, and dopamine responses. At 24 h post-EIMD, urinary 3-methylhistidine and the 3-methylhistidine/urinary creatinine ratio were lower in the GKK1 group than in the placebo group. No adverse changes were observed in clinical safety biomarkers. Conclusion: These findings suggest that GKK1 may be a safe nutritional strategy to facilitate functional recovery, attenuate secondary inflammatory responses and modulate urinary markers associated with muscle breakdown following muscle-damaging exercise in healthy young men.

## 1. Introduction

Exercise-induced fatigue (EIF) is a complex, multifactorial physiological phenomenon defined as a transient and reversible decline in the capacity to maintain physical performance, power output, or force generation under a specific exercise workload [1]. To accurately characterize this decline, fatigue must be distinguished into central fatigue, which involves an attenuation of central neuromuscular drive, and peripheral fatigue, which involves impairments in metabolic energy availability and excitation–contraction coupling. Its underlying mechanisms encompass several interconnected domains, including energy depletion, the accumulation of metabolic by-products, neuromuscular impairment, systemic inflammation, oxidative stress, and mitochondrial dysfunction [2]. Within this complex physiological framework, exercise-induced muscle damage (EIMD)—characterized by structural disruption to skeletal muscle fibers—acts as a pivotal factor that exacerbates fatigue severity and prolongs the recovery timeline [3]. Particularly when athletes engage in high-intensity or unaccustomed eccentric exercise, muscle damage occurs in two distinct phases. Primary damage involves the initial mechanical shear of sarcomeres and microtrauma to the z-discs during eccentric contraction. This mechanical disruption triggers secondary damage, which is characterized by a subsequent inflammatory cascade involving neutrophil and macrophage infiltration, reactive oxygen species production, and myofibrillar proteolysis [4]. Because nutritional interventions cannot physically prevent the initial mechanical shear, their protective mechanisms primarily target the attenuation and rapid resolution of this secondary damage phase, thereby mitigating the state of physical fatigue. The clinical manifestations of EIMD include delayed-onset muscle soreness (DOMS), swelling, pronounced deficits in muscle strength, and the leakage of intramuscular enzymes, such as creatine kinase, into the systemic circulation [5]. These symptoms typically peak at 24 to 48 h post-exercise, with functional impairment and soreness frequently persisting for 5 to 7 days [6]. The profound impact of such damage not only acutely compromises an athlete’s immediate training capacity but also, in the absence of timely and adequate recovery, substantially elevates the risk of subsequent overuse injuries. Therefore, mitigating the severity and duration of EIMD and DOMS has become a practical priority for maintaining training quality and accelerating return to play. To this end, a diverse range of strategies spanning scientific training modalities, compression garments, and nutritional interventions is commonly implemented to alleviate symptoms and facilitate the comprehensive recovery of neuromuscular function [7].

Dietary supplementation has emerged as a cornerstone strategy for optimizing functional support systems within modern athletic populations. Beyond the primary objective of enhancing exercise performance, these interventions serve to accelerate post-exercise recovery kinetics, preserve physiological homeostasis, and attenuate exercise-induced tissue damage, thereby ensuring the sustainability of long-term training adaptations [8]. Recent advancements in the ‘gut–muscle axis’ paradigm have elucidated the pivotal mechanistic role of the gut microbiota in modulating both EIF and EIMD, underscoring the clinical utility of microbiome-targeted nutritional strategies [9]. As a prominent intervention, probiotics not only modulate the microbial landscape to bolster energy metabolism and elevate fatigue thresholds but also exert multidimensional protective effects ranging from intestinal barrier reinforcement to systemic metabolic regulation [10]. Specifically, by fortifying intestinal tight junction integrity, probiotics effectively mitigate the translocation of lipopolysaccharides (LPS) into systemic circulation, thereby abating the exercise-induced systemic inflammatory cascade at its source [11]. Furthermore, preclinical evidence indicates that gut microbiota-derived short-chain fatty acids, specifically acetate, propionate, and butyrate, actively cross talk with skeletal muscle tissue. By binding to G protein-coupled receptors like GPR41 and GPR43 and acting as histone deacetylase inhibitors, these metabolites possess the theoretical capacity to regulate muscle protein synthesis, promote mitochondrial biogenesis, and suppress systemic inflammation. While these pathways present a highly promising theoretical framework, their definitive clinical translation to human exercise recovery remains to be fully elucidated. Concurrently, through the modulation of neuroendocrine pathways, probiotics optimize redox homeostasis by upregulating the expression of endogenous antioxidant enzymes, such as superoxide dismutase (SOD), thereby augmenting cellular defense mechanisms against oxidative stress [12]. This integrated effect spanning from intestinal barrier restoration to systemic metabolic fine-tuning facilitates the clearance of fatigue-related metabolic by-products and biomarkers, ultimately fostering a physiological milieu conducive to optimized energy utilization and heightened fatigue tolerance. Since clinical assessments frequently utilize explosive neuromuscular tests like the Countermovement Jump and Isometric Mid-Thigh Pull alongside glycolytic metabolic evaluations like the Wingate Anaerobic Test, it is crucial to recognize how probiotics support performance across these domains. Probiotic-driven changes in the gut environment can theoretically preserve central neuromuscular drive via the vagus nerve and neuroendocrine pathways, including dopamine and serotonin regulation, while simultaneously sustaining peripheral metabolic energy availability. This dual regulation attenuates the onset of EIF and expedites functional recovery following EIMD [9].

The genus *Lactobacillus* spp. constitutes a dominant taxonomic group of Gram-positive, non-motile, non-spore-forming, and acid-tolerant facultative anaerobes within the intestinal microecosystem [13]. While these microorganisms are widely recognized as probiotics exerting multifunctional physiological benefits, the literature presents conflicting evidence regarding their efficacy in athletic contexts, with several trials reporting no significant ergogenic or recovery benefits following supplementation. These discrepancies underscore that the ergogenic and protective efficacies of probiotics in sports nutrition are highly strain-specific [14]. For instance, regarding mucosal immune regulation, *Lactobacillus helveticus* Lafti L10 has been demonstrated to significantly enhance mucosal immune responses in elite athletes, thereby mitigating the risk of upper respiratory tract infections [15]. In terms of physical performance, *L. plantarum* TWK10 increases skeletal muscle mass, enhances muscle strength, and improves endurance capacity in both murine models and humans by optimizing energy metabolism and facilitating neuromuscular adaptation [16]. Concurrently, *L. plantarum* PS128 not only expedites exercise-capacity recovery following a half-marathon [17], but also effectively modulates the gut microbiota landscape in endurance athletes to attenuate muscle damage, oxidative stress, and systemic inflammatory responses induced by prolonged, high-intensity exertion [18]. Furthermore, the scope of microbial interventions extends beyond lactobacilli; a two-week co-ingestion of 20 g of casein and 1 billion CFU of *Bacillus coagulans* GBI-30, 6086 daily significantly reduces exercise-induced muscle damage and accelerates functional recovery in recreationally trained males [19]. Collectively, these findings highlight the profound strain-specific nature of probiotic efficacy in optimizing exercise recovery, immune modulation, and physical performance, thereby underscoring the critical necessity to empirically evaluate each novel candidate strain directly.

However, despite accumulating evidence supporting the potential of selected *L. plantarum* strains in attenuating exercise-induced fatigue, muscle damage, and inflammatory stress, the specific physiological rationale for selecting the GKK1 strain stems from its unique genomic background. Peer-reviewed genomic and bioinformatics analyses reveal that *L. plantarum* GKK1 harbors unique metabolic pathways and secondary metabolites, including predicted bacteriocins associated with robust immune modulation. Furthermore, the strain possesses a specific nitrate reductase region, with its high expression confirmed via qPCR, indicating its strong ability to produce nitric oxide for essential immune regulation and host defense. This is found alongside specific glucorhamnan-related gene clusters that actively stimulate interferon beta and interleukin 12 production [20]. These comprehensive features give GKK1 robust immunomodulatory and anti-inflammatory potential to combat secondary muscle damage. Nevertheless, robust empirical evidence regarding the functional recovery benefits of this specific probiotic in human trials remains limited [21]. To address this gap, the present randomized, double-blind, placebo-controlled trial evaluated the effects of four weeks of L. plantarum GKK1 supplementation on exercise-induced muscle damage, inflammatory and oxidative stress biomarkers, and recovery-related physical performance outcomes following a standardized muscle-damaging exercise protocol. Crucially, to comprehensively evaluate systemic recovery beyond localized muscle damage, this study incorporated the measurement of neuroendocrine and sympathoadrenal biomarkers. Assessing testosterone, cortisol, and catecholamines allows for a precise evaluation of the anabolic-to-catabolic balance and the systemic stress response, providing deeper insights into the holistic recovery kinetics. To rigorously evaluate myofibrillar proteolysis, this study incorporated the measurement of urinary 3-methylhistidine. Because 3-methylhistidine is a highly specialized and direct biomarker of skeletal muscle protein breakdown, it provides complementary evidence to traditional and highly variable serum markers like creatine kinase, offering a precise index of structural damage resolution.

## 2. Materials and Methods

### 2.1. Participants

A total of 48 healthy, sedentary adult male participants with no regular exercise habits (engaging in less than 1 h of physical activity per week) were recruited and enrolled in this study. Baseline physical activity levels were verified via questionnaire to ensure homogeneity and minimize confounding from the repeated bout effect. Participants were excluded if they were taking medications, antioxidant or anti-inflammatory drugs, amino acid supplements, chicken essence products, or other nutritional supplements that could influence exercise performance or biochemical outcomes. Furthermore, to ensure a stable baseline gut microbiome, participants were strictly excluded if they had consumed any antibiotic medications or commercial probiotic supplements within the four weeks prior to enrollment. The experimental protocol was reviewed and formally approved by the Institutional Review Board of Landseed International Hospital, Taiwan (Protocol No.: IRB-24-034-A2; approval date: 30 May 2024). All investigative procedures were executed in strict accordance with the ethical tenets delineated in the Declaration of Helsinki. Written informed consent was obtained from all participants before enrollment. The clinical trial was retrospectively registered at ClinicalTrials.gov under the identifier NCT06893549. As an investigator-initiated academic trial, the study protocol and all primary outcomes were strictly pre-approved by the Institutional Review Board on 30 May 2024, prior to the official commencement of participant recruitment on 24 September 2024. The trial record was subsequently submitted to the international registry on 17 March 2025, and publicly posted on 25 March 2025. This sequence ensured complete local regulatory compliance and locked all methodological parameters prior to enrollment, precluding any possibility of post hoc outcome modification. The study completion report was reviewed and approved by the Institutional Review Board on 25 July 2025.

### 2.2. Study Design

This study was conducted as a randomized, double-blind, placebo-controlled, parallel-group clinical trial. Participants were randomly assigned to either the placebo group (*n* = 24) or the GKK1 group (*n* = 24) using a computer-generated randomization sequence. Allocation concealment was strictly ensured by an independent researcher using sequentially numbered, opaque, sealed envelopes. The *L. plantarum* GKK1 supplement used in this study was manufactured by Grape King Bio Ltd. (Taoyuan, Taiwan) in a production facility operating under ISO 22000: 2018 [22] and Hazard Analysis and Critical Control Point (HACCP) food safety management systems. Quality control testing was conducted by the manufacturer’s internal Quality Control (QC) laboratories, which are accredited by the Taiwan Accreditation Foundation (TAF) and operate in accordance with ISO/IEC 17025 standards [23]. Each production batch underwent routine quality control testing, including viable cell count (CFU), microbiological testing, physicochemical analyses, and food safety hazard assessments according to the manufacturer’s quality specifications. Only samples meeting all predefined quality criteria were released for use in this study. The GKK1 group ingested two *L. plantarum* GKK1 probiotic capsules daily for 28 consecutive days, providing approximately 1000 mg per day. Each capsule contained 5.0 × 10^10^ CFU, corresponding to a guaranteed total daily dose of 1.0 × 10^11^ CFU with bacterial viability maintained until the end of the trial shelf life. The placebo group received two identical capsules daily composed of microcrystalline alpha-cellulose, magnesium stearate, and silicon dioxide, matched to the active supplement in appearance, size, taste, and weight to maintain blinding. Supplementation compliance was rigorously monitored through a daily dietary log and weekly capsule return counts, with all participants achieving a compliance rate greater than 91.2%. Baseline anthropometric characteristics were similar between groups. Mean age was 22.3 ± 1.9 years in the placebo group and 22.3 ± 2.2 years in the GKK1 group. Mean height was 177.2 ± 6.7 cm and 173.9 ± 4.7 cm, respectively, and mean body weight was 73.9 ± 7.8 kg and 70.0 ± 7.5 kg, respectively (Table 1). Following the 28-day supplementation period, all participants underwent the standardized EIMD induction protocol. Outcome measures were evaluated at baseline (pre-EIMD) and at 3, 24, and 48 h post-EIMD.

During the intervention period, participants were instructed to maintain their usual dietary habits and avoid nutritional supplements. Participants were also instructed to avoid alcohol intake and anti-inflammatory or antioxidant medications for one week before exercise testing and blood sampling. Before exercise testing, participants fasted for at least 12 h and avoided strenuous physical activity for three days. During all testing sessions, participants were provided with standardized water intake to maintain euhydration and minimize exercise-induced systemic fluid shifts.

### 2.3. Dietary Assessment

Dietary intake was recorded before and after the intervention to determine whether energy and macronutrient intake differed between groups or changed during the study. During the baseline screening, nutritional assessments confirmed that all enrolled participants maintained a habitual omnivorous diet. This ensured that profound variations in baseline gut microbiota composition, typically observed between omnivorous and vegan or vegetarian populations, did not confound the physiological responses to the probiotic intervention. Participants recorded three days of dietary intake using photographs and uploaded the dietary records to the Cofit application. Energy and macronutrient intake were analyzed by a registered dietitian using Cofit Pro software (Version 5.9.8, Cofit Healthcare Inc., Taipei, Taiwan). To minimize dietary confounding for urinary 3-methylhistidine analysis, participants were instructed to maintain a standardized daily protein intake. The dietitian verified through the Cofit records that total protein and meat consumption remained consistent and balanced between the two groups during the critical testing windows.

### 2.4. Body Composition

Body composition metrics including body weight, body mass index (BMI), lean body mass, and body fat percentage were systematically evaluated using a multifrequency bioelectrical impedance analyzer (BIA; InBody 570, InBody Co., Ltd., Seoul, Republic of Korea). To ensure data integrity and minimize confounding physiological variations, participants underwent assessments in the morning following an overnight fast of at least 8 h. Prior to electrode contact, the palms and soles of each participant were meticulously cleansed. Measurements were performed within a 60 s timeframe across a multifrequency spectrum (1, 5, 50, 260, 500, and 1000 kHz). During the assessment, participants stood barefoot on the foothold electrodes and gripped the sensing handrails in accordance with the manufacturer’s guidelines. A standardized testing posture was strictly maintained, characterized by holding the upper limbs at a 30° abduction angle relative to the trunk, while remaining stationary and silent throughout the scanning sequence. These BIA assessments were longitudinally performed at baseline, at bi-weekly intervals during the trial, and immediately upon the completion of the 28-day intervention.

### 2.5. Exercise-Induced Muscle Damage Protocol

To systematically induce muscle fatigue and exercise-induced muscle damage (EIMD), participants underwent a standardized, high-intensity plyometric protocol consisting of 100 maximal vertical jumps, adapted from validated methodologies establishing post-exercise recovery responses [24]. The chronological architecture of the protocol comprised 10 discrete sets of 10 consecutive repetitions. Within each set, individual jumps were executed at a strict cadence of one repetition every 4 s, followed by a 90 s passive rest interval between successive sets. Biomechanical standardization across all subjects was rigorously maintained by strictly monitoring and requiring participants to flex their knee joints to a 90° angle during the eccentric squat phase of each repetition. Because the eccentric deceleration phase is the primary driver of muscle damage, strictly enforcing this 90° depth ensured standardized mechanical strain across all participants, regardless of fatigue-induced declines in concentric jump height. To ensure the preservation of maximal mechanical output, each participant’s baseline maximum vertical jump height was visually marked as a target based on their individual height and arm length. This target served as a visual cue, and investigators provided standardized verbal encouragement throughout the protocol to stimulate maximal volitional effort for every repetition, preventing systematic pacing as fatigue accumulated. Although knee flexion depth, visual targets, cadence, and verbal encouragement were standardized, actual jump height, landing velocity, eccentric force, and mechanical work across all 100 repetitions were not continuously quantified. Therefore, equivalent mechanical loading between participants and groups cannot be definitively confirmed.

### 2.6. Countermovement Jump Test

To evaluate lower-limb maximal strength, explosive power, and velocity characteristics, countermovement jump (CMJ) assessments were conducted longitudinally at baseline (pre-EIMD) as well as at 3, 24, and 48 h post-EIMD [25]. Kinematic and kinetic data were acquired synchronously using a 10-camera motion capture system (Vicon T40, Oxford Metrics Ltd., Oxford, UK) sampling at 200 Hz and a portable piezoelectric force platform (Kistler 9260AA/9281B, Kistler Instruments AG, Winterthur, Switzerland) sampling at 1000 Hz. Prior to each testing sequence, participants completed a standardized 5 min dynamic warm-up on a cycle ergometer followed by two submaximal practice jumps. Subsequently, the force platform was calibrated relative to each participant’s static body mass to allow for proper weight normalization. For the standardization of biomechanical execution, participants stood barefoot on the force plate with hands fixed on their hips to eliminate upper-limb propulsive contribution, except for the tracking of a reflective marker affixed to the middle fingertip of the dominant hand. Upon command, participants executed a rapid eccentric countermovement until reaching a 90° knee flexion angle, followed immediately by a maximal-effort vertical jump. Each participant performed three valid trials at each designated time point, with the mean values utilized for subsequent statistical analysis. Kinetic and kinematic parameters including the rate of force development (RFD), peak power, and vertical jump height were systematically derived via a customized routine developed in MATLAB (R2017b, MathWorks, Natick, MA, USA).

### 2.7. Isometric Mid-Thigh Pull Test

To assess force-time characteristics, isometric mid-thigh pull (IMTP) assessments were systematically implemented at baseline (pre-EIMD) as well as at 3, 24, and 48 h post-EIMD. Kinetic data were quantified utilizing a customized IMTP testing apparatus (IMTP Rack, Kairos Strength, Murphy, CA, USA) integrated with a portable force platform system (Kistler 9260AA/9287BA, Kistler Instruments AG, Winterthur, Switzerland). Prior to formal data collection, participants completed a standardized 5 min dynamic warm-up on a cycle ergometer and underwent a standardized familiarization protocol incorporating submaximal pulls to ensure proper execution mechanics. During testing, participants stood barefoot on the force plates with a shoulder-width stance, a neutral spine, and an upright torso. The immovable barbell was precisely positioned at the midpoint of the thighs, with hip and knee extension angles strictly standardized at 140° using a goniometer. Once a stable weighing cycle and symmetrical force baseline were verified via real-time force traces, participants were instructed to exert maximal volitional upward force against the bar for a duration of 3 to 5 s. Each participant performed three maximal trials separated by a 2 min passive recovery interval to minimize neuromuscular fatigue and minimize postural confounding. The derived kinetic parameters comprising absolute relative peak force (normalized to body mass, N/kg) and peak rate of force development (pRFD) were extracted and processed for subsequent statistical analyses [26].

### 2.8. Wingate Anaerobic Test

To characterize anaerobic capacity and neuromuscular fatigue resistance, a classic 30 s Wingate Anaerobic Test (WAnT) was implemented longitudinally at baseline (pre-EIMD) as well as at 3, 24, and 48 h post-EIMD [27]. All testing sequences were executed utilizing a digitally controlled friction-belt cycle ergometer (Monark 894E, Monark Exercise AB, Vansbro, Sweden). Ergonomic parameters including saddle height and handlebar positioning were customized to each participant’s preference during initial profiling and strictly replicated across all subsequent testing sessions, with toe clips secured to prevent pedaling slippage. To eliminate learning effects and prevent energy-conserving pacing strategies, all participants completed a standardized familiarization trial on the cycle ergometer prior to the formal testing phase. Prior to the supramaximal evaluation on formal testing days, participants underwent a standardized 5 min warm-up at a baseline power output of approximately 50 W, interspersed with two brief 3 s preparatory sprints under a light uncoupling load (3% of body weight) to achieve post-activation neuromuscular potentiation. Following the warm-up, participants were instructed to accelerate to maximal pedaling cadence. To strictly prevent pacing, participants were explicitly commanded to exert an immediate and absolute maximum effort. Immediately upon reaching an optimal velocity threshold of 120 rpm, an external resistive load meticulously calculated at 7.5% of the participant’s static body mass was automatically deployed onto the friction belt. Participants maintained a true maximal, all-out sprint effort throughout the remaining 30 s epoch against this constant resistance. The derived anaerobic power metrics comprising relative peak power (RPP, W/kg), relative mean power (RMP, W/kg), and the fatigue index (FI, %) were captured and computed for subsequent kinetic profiling.

### 2.9. Blood Biochemical Analysis

Blood samples were collected before supplementation for baseline safety assessment and at pre-EIMD, 3 h, 24 h, and 48 h post-EIMD for mechanistic and recovery-related biomarkers. To tightly control for diurnal neuroendocrine rhythms, all blood collections were strictly conducted in the morning between 08:00 and 12:00. Furthermore, each participant’s individual collection time was held constant across all longitudinal testing days. Serum was obtained by centrifugation at 1500× *g* for 15 min at 4 °C. Liver function, renal function, lipid profiles, and glucose were assessed using an automated biochemical analyzer (Hitachi 717, Hitachi, Tokyo, Japan). Safety indicators included aspartate aminotransferase, alanine aminotransferase, blood urea nitrogen, creatinine, uric acid, total cholesterol, triglycerides, high-density lipoprotein cholesterol, low-density lipoprotein cholesterol, and glucose.

Muscle damage and inflammatory biomarkers included CK, myoglobin, and high-sensitivity CRP (hs-CRP). CK and hs-CRP were analyzed using an AU 5820 autoanalyzer (Beckman Coulter Inc., Brea, CA, USA), and myoglobin was analyzed using a DXI 800 autoanalyzer (Beckman Coulter Inc., Brea, CA, USA). Oxidative stress was assessed using a TBARS Assay Kit (No. 10009055, Cayman Chemical Company, Ann Arbor, MI, USA). Endocrine and stress-related biomarkers included testosterone, cortisol, growth hormone, adrenaline, noradrenaline, and dopamine.

### 2.10. Urinary 3-Methylhistidine Analysis

Urine samples were collected to assess 3-methylhistidine and urinary creatinine concentrations. Prior to ratio calculation, absolute urinary creatinine values were screened to ensure no samples were subjected to extreme dilution. The 3-methylhistidine/urinary creatinine ratio was calculated to normalize urinary 3-methylhistidine excretion and was used as an indicator of myofibrillar proteolysis.

### 2.11. Statistical Analysis

All experimental data are expressed as mean ± standard deviation (SD). Statistical analyses were performed using IBM SPSS Statistics version 24.0 (IBM Corp., Armonk, NY, USA). Sample size was determined a priori using G*Power software (Version 3.1). Assuming a moderate effect size (f = 0.25) for a two-way repeated-measures ANOVA with an alpha level of 0.05 and a beta power of 0.80, a minimum of 36 participants was required. We recruited 48 participants to account for potential dropouts and ensure robust statistical power. Statistical analyses were performed using IBM SPSS Statistics version 24.0 (IBM Corp., Armonk, NY, USA). Prior to parametric testing, the normality of the data distribution was verified using the Shapiro–Wilk test, and the assumption of sphericity was assessed using Mauchly’s test. To appropriately resolve baseline imbalances, an Analysis of Covariance (ANCOVA) was applied for specific biomarkers (e.g., dopamine), utilizing the pre-EIMD values as a covariate. To evaluate the effects of the intervention across multiple longitudinal time points, a two-way repeated measures analysis of variance (RM ANOVA) was used to determine the main effects of treatment group and time, as well as the group-by-time interaction. When a significant main effect or interaction was detected, Bonferroni-adjusted post hoc pairwise comparisons were applied to strictly control for Type I errors associated with multiple testing. Statistical significance was set a priori at *p* < 0.05.

## 3. Results

### 3.1. Participant Dietary Intake, Body Composition and Safety-Related Biochemical Parameter

Forty-eight healthy male participants completed the study and were included in the analysis. No significant between-group differences were observed in dietary carbohydrate, protein, fat, or total energy intake before or after the intervention (Table S1). Body weight, body mass index, lean body mass, and body fat percentage were not significantly different between groups and did not significantly change after four weeks of supplementation (Table S2). Also, GKK1 supplementation did not adversely affect liver function, renal function, blood glucose, or lipid profiles. These findings indicate that GKK1 supplementation was well tolerated in healthy adult men (Table S3).

### 3.2. Effects of GKK1 Supplementation on Handgrip Strength

Handgrip strength was assessed before and after the intervention. Between-group differences were not significant. The placebo group showed numerical decreases in left and right handgrip strength after the intervention, whereas the GKK1 group showed numerical increases; however, these changes should be interpreted cautiously because no significant between-group differences were detected (Table 2).

### 3.3. Effects of GKK1 Supplementation on Countermovement Jump Recovery

Prior to the execution of the EIMD protocol, baseline homogeneity was confirmed, as no significant differences were observed between the placebo and GKK1 cohorts across all primary metrics, including absolute rate of force development (RFD) (*p* = 0.861), relative force peak (*p* = 0.942), and jump height (*p* = 0.961). A two-way repeated-measures ANOVA revealed significant group-by-time interactions across all three performance parameters: RFD [*F*(3, 138) = 3.419, *p* = 0.024, *η*^2^ = 0.069], relative force peak [*F*(3, 138) = 13.022, *p* < 0.001, *η*^2^ = 0.221], and jump height [*F*(3, 138) = 4.320, *p* = 0.006, *η*^2^ = 0.086] (Table 3). Additionally, a significant main effect of time was universally detected (all *p* < 0.001, *η*^2^ ranging from 0.367 to 0.831), confirming that the 100-repetition vertical jump protocol successfully induced neuromuscular fatigue at 3, 24, and 48 h post-exercise.

**Figure 1:**
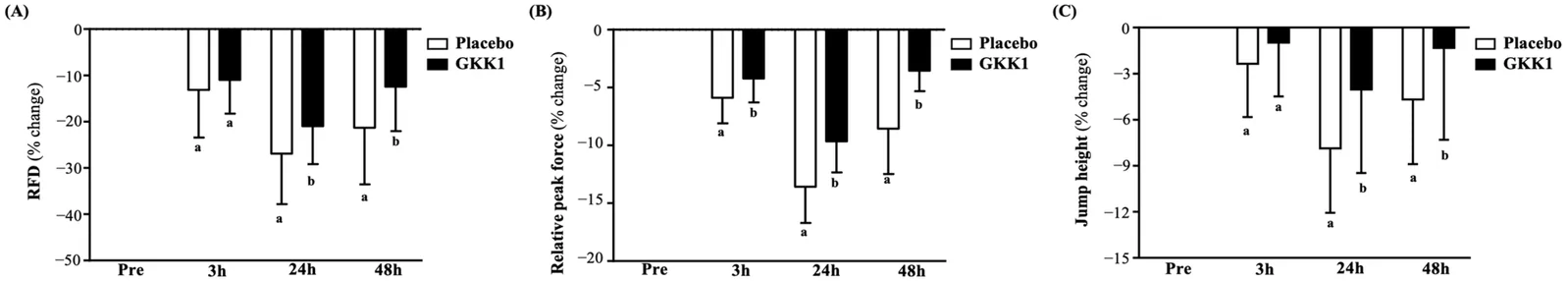
Effects of 4 weeks of GKK1 supplementation on percentage changes in CMJ performance: (**A**) RFD, (**B**) relative peak force, and (**C**) jump height. Data are expressed as mean ± SD (*n* = 24 per group). Different superscript letters indicate significant differences between groups at the same time point (*p* < 0.05). RFD, rate of force development.

Subsequent post hoc pairwise comparisons indicated the recovery-supporting effect of the GKK1 intervention. While absolute RFD decrements were statistically comparable between groups at 3 h post-EIMD (*p* = 0.407), the GKK1 cohort demonstrated significantly superior absolute RFD compared to the placebo group at 24 h (*p* = 0.033, 95% CI [0.06 to 1.32], Cohen’s *d* = 0.635) and 48 h post-EIMD (*p* = 0.001, 95% CI [0.42 to 1.54], Cohen’s *d* = 1.036) (Table 3). When normalized as a percentage change from baseline, the GKK1 group exhibited a significantly attenuated relative loss in RFD at 24 h (−20.97 ± 8.22% vs. −26.91 ± 10.92%, *p* = 0.039, 95% CI [0.33 to 11.56], Cohen’s *d* = 0.615) and an accelerated recovery trajectory by 48 h (−12.43 ± 9.60% vs. −21.33 ± 12.20%, *p* = 0.007, 95% CI [2.53 to 15.28], Cohen’s *d* = 0.810) (Figure 1A).

Similar protective profiles were observed in maximal force generation capabilities. When examining the relative percentage change, the GKK1 group exhibited significantly less performance decrement compared to the placebo group as early as 3 h post-EIMD (−4.22 ± 2.07% vs. −5.89 ± 2.20%, *p* = 0.009, 95% CI [0.44 to 2.92], Cohen’s *d* = 0.786). This protective attenuation was sustained and further amplified at 24 h (−9.65 ± 2.69% vs. −13.58 ± 3.13%, *p* < 0.001, 95% CI [2.24 to 5.62], Cohen’s *d* = 1.348). By 48 h post-EIMD, the GKK1 group not only demonstrated a significantly higher absolute relative force peak (*p* = 0.048, 95% CI [0.01 to 1.63], Cohen’s *d* = 0.580) (Table 3), but also maintained a remarkably lower percentage deficit (−3.54 ± 1.78% vs. −8.56 ± 3.92%, *p* < 0.001, 95% CI [3.26 to 6.79], Cohen’s *d* = 1.652) (Figure 1B).

Although absolute jump height values did not reach intergroup statistical significance due to inherent individual biomechanical variations, the percentage change data unveiled distinct recovery kinetics. Analysis indicated that GKK1 supplementation significantly mitigated the acute loss of jump height at 24 h post-EIMD (−4.03 ± 5.44% vs. −7.86 ± 4.21%, *p* = 0.009, 95% CI [1.01 to 6.66], Cohen’s *d* = 0.788). Furthermore, at 48 h post-EIMD, the GKK1 group exhibited a significantly enhanced restoration of jumping capacity, with a minimal residual deficit compared to the placebo group (−1.32 ± 5.99% vs. −4.68 ± 4.21%, *p* = 0.029, 95% CI [0.36 to 6.37], Cohen’s *d* = 0.650) (Figure 1C).

### 3.4. Effects of GKK1 Supplementation on Isometric Mid-Thigh Pull Performance

Prior to the execution of the EIMD protocol, baseline homogeneity was confirmed, as no significant differences were observed between the placebo and GKK1 cohorts across primary kinetic metrics, including relative peak force (*p* = 0.735) and peak rate of force development (pRFD, *p* = 0.835). A two-way repeated-measures ANOVA revealed highly significant group-by-time interactions for both performance parameters: relative peak force [*F*(3, 138) = 15.513, *p* < 0.001, *η*^2^ = 0.252] and pRFD [*F*(3, 138) = 11.539, *p* < 0.001, *η*^2^ = 0.201] (Table 4). Additionally, a main effect of time was universally detected (all *p* < 0.001), confirming that the plyometric protocol successfully induced neuromuscular fatigue at 3, 24, and 48 h post-exercise.

**Figure 2:**
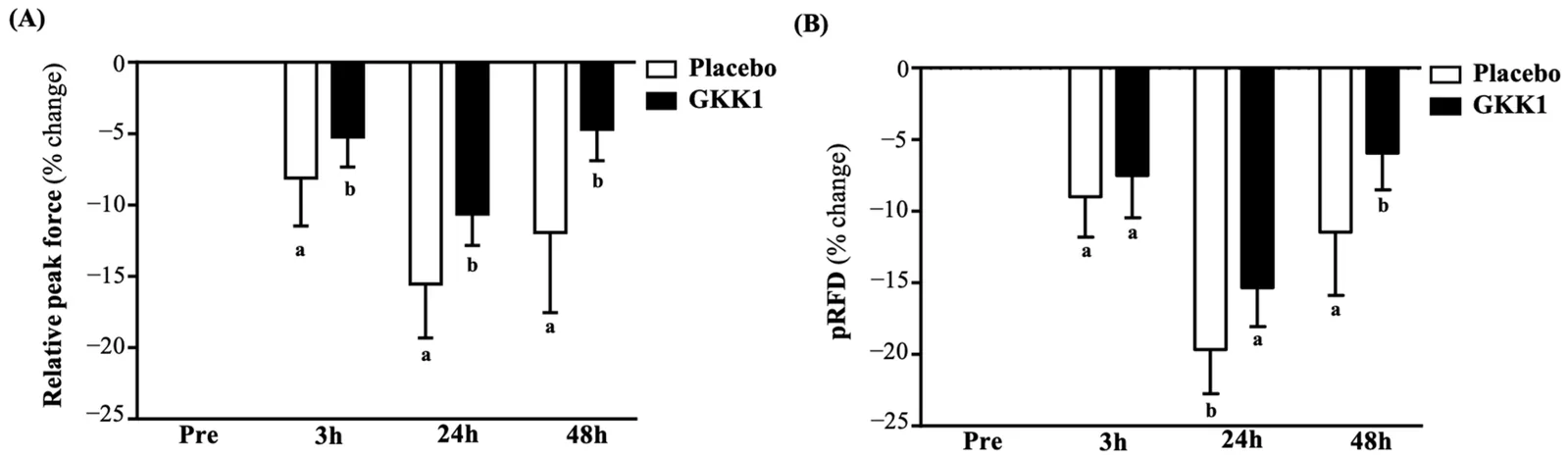
Effects of 4 weeks of GKK1 supplementation on percentage changes in IMTP performance: (**A**) relative peak force and (**B**) peak RFD. Data are expressed as mean ± SD (*n* = 24 per group). Different superscript letters indicate significant differences between groups at the same time point (*p* < 0.05). RFD, rate of force development.

Subsequent post hoc pairwise comparisons elucidated the robust restorative efficacy of the GKK1 intervention on maximal force generation. When examining the percentage change from baseline, the GKK1 group exhibited significantly less performance decrement compared to the placebo group as early as 3 h post-EIMD (−5.23 ± 2.11% vs. −8.12 ± 3.35%, *p* = 0.001, 95% CI [1.30 to 4.46], Cohen’s *d* = 1.029). This protective attenuation was sustained and further amplified at 24 h, where the GKK1 group demonstrated both a significantly higher absolute relative peak force (*p* = 0.016, 95% CI [0.18 to 1.56], Cohen’s *d* = 0.720) (Table 4) and a markedly lower percentage deficit (−10.63 ± 2.20% vs. −15.54 ± 3.78%, *p* < 0.001, 95% CI [3.16 to 6.66], Cohen’s *d* = 1.587). By 48 h post-EIMD, the GKK1 group maintained a significantly superior absolute relative peak force (*p* = 0.002, 95% CI [0.51 to 1.99], Cohen’s *d* = 0.950) (Table 4) and an accelerated recovery trajectory in percentage change (−4.70 ± 2.19% vs. −11.94 ± 5.60%, *p* < 0.001, 95% CI [4.83 to 9.65], Cohen’s *d* = 1.700) (Figure 2A).

Similar protective profiles were observed in the maximal rate of force development. Although absolute pRFD values did not reach intergroup statistical significance at 24 h post-EIMD due to inherent interindividual variations (*p* = 0.105), normalizing the data unveiled distinct recovery kinetics. Analysis of the percentage change from baseline indicated that GKK1 supplementation significantly mitigated the acute loss of pRFD at 24 h (−15.34 ± 2.72% vs. −19.66 ± 3.09%, *p* < 0.001, 95% CI [2.66 to 5.96], Cohen’s *d* = 1.483). Furthermore, at 48 h post-EIMD, the GKK1 group exhibited a significantly enhanced restoration of explosive capacity, reflected in both a higher absolute pRFD (*p* = 0.037, 95% CI [42 to 906], Cohen’s *d* = 0.621) (Table 4) and a minimal residual percentage deficit compared to the placebo group (−5.96 ± 2.55% vs. −11.47 ± 4.42%, *p* < 0.001, 95% CI [3.47 to 7.55], Cohen’s *d* = 1.526) (Figure 2B).

### 3.5. Effects of GKK1 Supplementation on Wingate Anaerobic Power

Prior to the execution of the supramaximal anaerobic cycling protocol, baseline homogeneity was confirmed, with no significant differences observed between the placebo and GKK1 cohorts across all primary variables, including relative mean power (*p* = 0.153), relative peak power (*p* = 0.862), and fatigue index (*p* = 0.943). A two-way repeated-measures ANOVA revealed a highly significant group-by-time interaction for relative mean power [*F*(3, 138) = 10.579, *p* < 0.001, *η*^2^ = 0.187] and a marginally significant interaction for relative peak power [*F*(3, 138) = 2.640, *p* = 0.060, *η*^2^ = 0.054] (Table 5). A main effect of time was universally detected across all metrics (all *p* < 0.001), confirming that the exercise protocol successfully induced systemic and neuromuscular fatigue at 3, 24, and 48 h post-exercise.

**Figure 3:**
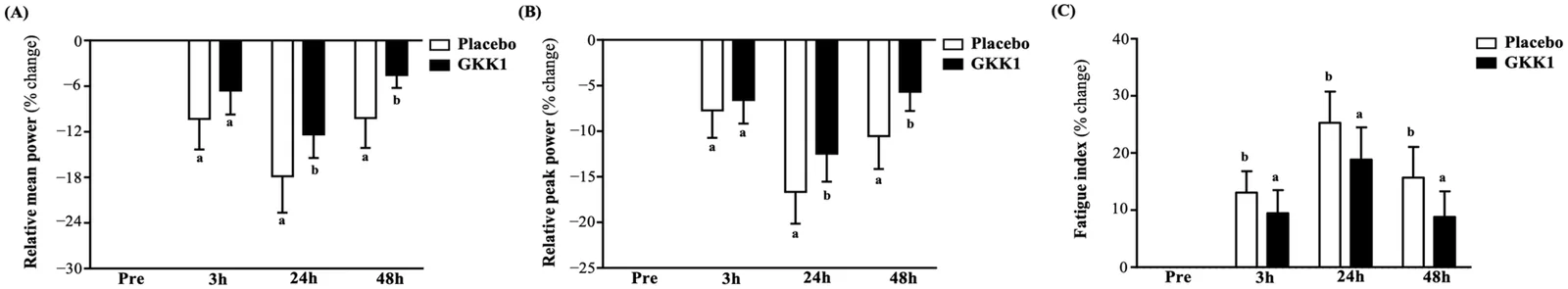
Effects of 4 weeks of GKK1 supplementation on percentage changes in Wingate anaerobic test performance: (**A**) relative mean power, (**B**) relative peak power, and (**C**) fatigue index. Data are expressed as mean ± SD (*n* = 24 per group). Different superscript letters indicate significant differences between groups at the same time point (*p* < 0.05).

Given that the overall group-by-time interaction for the fatigue index and relative peak power did not achieve strict statistical significance, subsequent pairwise comparisons were conducted as exploratory analyses. These exploratory analyses indicated that GKK1 supplementation may potentially support recovery of sustained anaerobic capacity. The GKK1 group demonstrated significantly higher absolute relative mean power than the placebo group at 24 h (*p* < 0.001, Cohen’s *d* = 1.094) and 48 h post-EIMD (*p* < 0.001, Cohen’s *d* = 1.156) (Table 5). When normalized as percentage change from baseline, the GKK1 group exhibited a smaller loss in mean power at 24 h (−12.35 ± 3.09% vs. −17.86 ± 4.78%, *p* < 0.001, 95% CI [3.18 to 7.84], Cohen’s *d* = 1.371) and a faster recovery by 48 h (−4.55 ± 1.68% vs. −10.19 ± 3.94%, *p* < 0.001, 95% CI [3.88 to 7.40], Cohen’s *d* = 1.861) relative to placebo (Figure 3A).

Similar patterns were observed for maximal anaerobic bursts. Although absolute relative peak power did not reach between-group significance at 24 h post-EIMD (*p* = 0.051), percentage change analysis showed that GKK1 supplementation attenuated the loss of peak power at 24 h (−12.48 ± 3.06% vs. −16.65 ± 3.49%, *p* < 0.001, 95% CI [2.27 to 6.07], Cohen’s d = 1.270). At 48 h post-EIMD, the GKK1 group showed higher absolute relative peak power (*p* = 0.025, Cohen’s d = 0.667) (Table 5) and a smaller residual percentage deficit than the placebo group (−5.69 ± 2.10% vs. −10.54 ± 3.60%, *p* < 0.001, 95% CI [3.15 to 6.55], Cohen’s d = 1.646) (Figure 3B).

In the context of anaerobic performance, an attenuated increase in the fatigue index denotes superior fatigue resistance. Although the overall group by time interaction did not achieve statistical significance (*p* = 0.140), planned pairwise comparisons of the normalized data revealed between-group differences in fatigue accumulation. The GKK1 group demonstrated a significantly lower percentage spike in fatigue index compared to the placebo group at 24 h post-EIMD (18.84 ± 5.66% vs. 25.28 ± 5.49%, *p* < 0.001, 95% CI [−9.66 to −3.24], Cohen’s *d* = 1.154). This lower fatigue index elevation was sustained at 48 h, where the GKK1 group exhibited an expedited return toward baseline, maintaining a significantly lower fatigue index elevation relative to the placebo cohort (8.80 ± 4.49% vs. 15.69 ± 5.36%, *p* < 0.001, 95% CI [−9.73 to −4.03], Cohen’s *d* = 1.394) (Figure 3C).

### 3.6. Effects of GKK1 Supplementation on Assessment of Muscle Damage and Inflammatory Biomarkers

Prior to the EIMD protocol, baseline homogeneity was confirmed across the assessed systemic biomarkers (all *p* > 0.05). A two-way repeated-measures ANOVA indicated significant group-by-time interactions for serum CK, myoglobin, CRP, and TBARS (all *p* < 0.001, *η*^2^ ranging from 0.479 to 0.646), with significant main effects of time (all *p* < 0.001), supporting successful induction of muscle damage, inflammation, and lipid peroxidation after the EIMD protocol.

**Figure 4:**
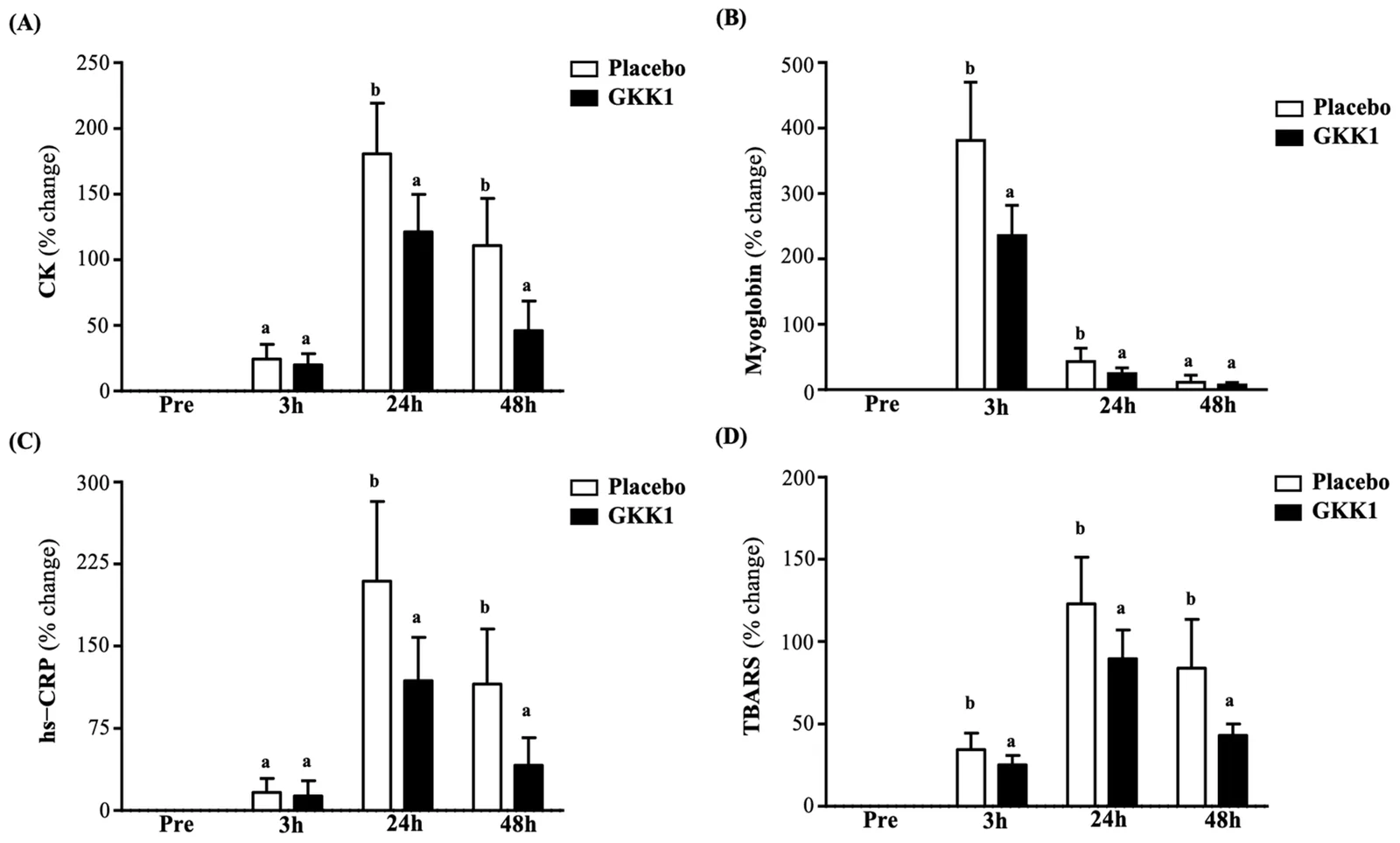
Effects of 4 weeks of GKK1 supplementation on percentage changes in blood biomarkers: (**A**) CK, (**B**) myoglobin, (**C**) hs-CRP, and (**D**) TBARS. Data are expressed as mean ± SD (*n* = 24 per group). Different superscript letters indicate significant differences between groups at the same time point (*p* < 0.05). CK, creatine kinase; hs-CRP, high-sensitivity C-reactive protein; TBARS, thiobarbituric acid reactive substances.

The GKK1 intervention attenuated structural muscle damage. Following this acute efflux, GKK1 significantly blunted the delayed serum CK elevations, accelerating the return of sarcolemmal integrity at 24 h (121.30 ± 28.41% vs. 180.74 ± 38.42%, *p* < 0.001, 95% CI [−79.07 to −39.81], Cohen’s *d* = 1.759) and maintaining significantly lower absolute CK levels through 48 h (*p* < 0.001, Cohen’s *d* = 2.167) (Table 6 and Figure 4A). GKK1 attenuated the early-phase relative myoglobin spike at 3 h post-EIMD (235.41 ± 46.33% vs. 381.08 ± 88.94%, *p* < 0.001, 95% CI [−186.87 to −104.47], Cohen’s *d* = 2.054) (Figure 4B). These data suggest that GKK1 supplementation may help reduce the progressive secondary efflux of intracellular markers during post-exercise recovery.

The attenuation of structural muscle damage subsequently modulated secondary inflammatory and oxidative cascades. Normalized percentage data revealed that GKK1 reduced the relative CRP inflammatory burden at 24 h compared to the placebo (118.72 ± 39.32% vs. 209.45 ± 72.90%, *p* < 0.001, 95% CI [−124.76 to −56.70], Cohen’s *d* = 1.549) and maintained significantly lower absolute CRP levels through 48 h (*p* < 0.001, Cohen’s *d* = 1.873) (Table 6 and Figure 4C). Concurrently, lipid peroxidation was systematically mitigated, with GKK1 exhibiting a lower relative TBARS oxidative spike at 48 h (43.20 ± 6.88% vs. 84.01 ± 29.46%, *p* < 0.001, 95% CI [−53.24 to −28.38], Cohen’s *d* = 1.910), alongside significantly lower absolute TBARS concentrations as early as 24 h post-EIMD (*p* < 0.001, Cohen’s *d* = 2.303) (Table 6 and Figure 4D).

### 3.7. Effects of GKK1 Supplementation on Neuroendocrine and Metabolic Profiling

Prior to the EIMD protocol, baseline homogeneity was confirmed across hormonal and neurotransmitter biomarkers (*p* > 0.05, except for baseline dopamine). A two-way repeated-measures ANOVA indicated significant group-by-time interactions for testosterone, cortisol, HGH, adrenaline, noradrenaline, and dopamine (all *p* < 0.01, *η*^2^ ranging from 0.095 to 0.825), accompanied by significant main effects of time. These results suggest that GKK1 supplementation was associated with altered neuroendocrine and sympathoadrenal responses following high-intensity mechanical stress.

**Figure 5:**
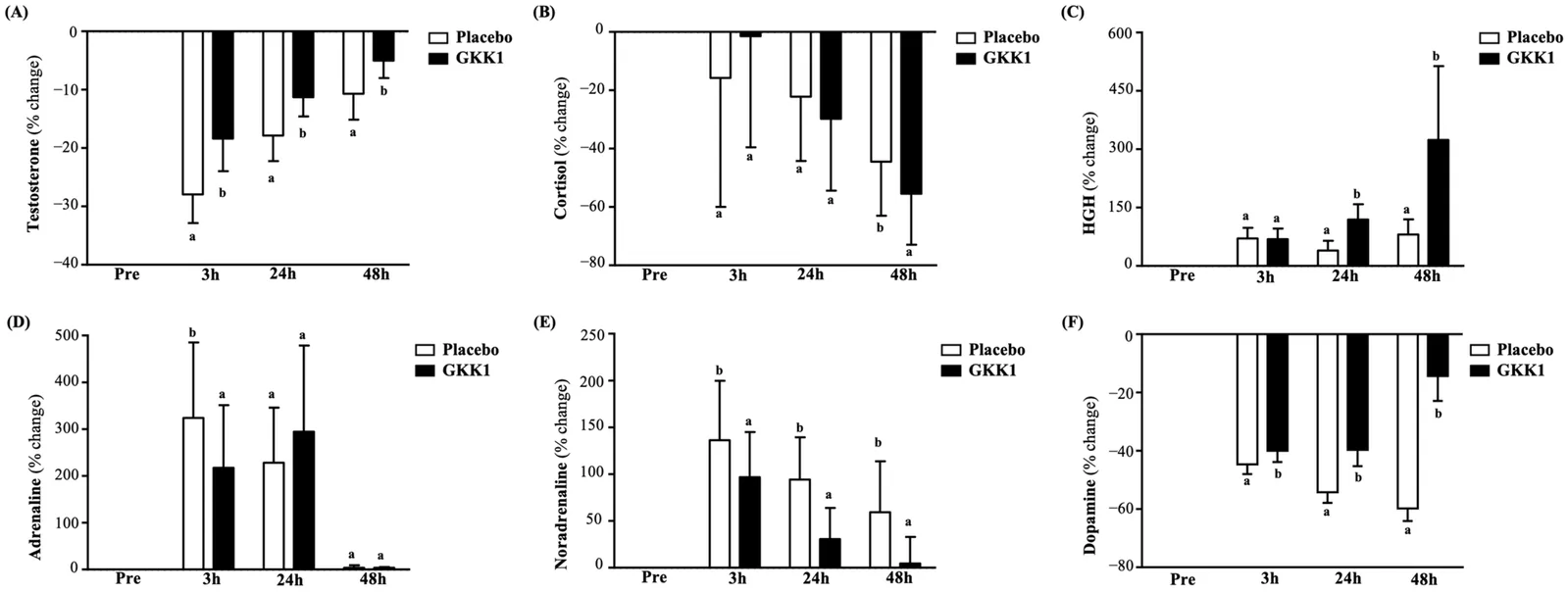
Effects of 4 weeks of GKK1 supplementation on percentage changes in blood biomarkers: (**A**) testosterone, (**B**) cortisol, (**C**) HGH, (**D**) adrenaline, (**E**) noradrenaline, and (**F**) dopamine. Data are expressed as mean ± SD (*n* = 24 per group). Different superscript letters indicate significant differences between groups at the same time point (*p* < 0.05). HGH, human growth hormone.

GKK1 supplementation was associated with a more favorable anabolic–catabolic profile after EIMD. Although serum testosterone decreased after EIMD in both groups, the GKK1 group maintained higher absolute testosterone levels at 3 h (*p* = 0.002, 95% CI [0.21 to 0.75], Cohen’s *d* = 1.011) and 24 h post-EIMD (*p* = 0.017, 95% CI [0.07 to 0.53], Cohen’s *d* = 0.713) (Table 6 and Figure 5A). Although the overall group main effect was not significant, a significant group-by-time interaction was observed for cortisol (*p* = 0.008). The GKK1 group showed lower cortisol levels than the placebo group at 48 h post-EIMD (*p* = 0.023, 95% CI [−2.73 to −0.29], Cohen’s *d* = 0.680) (Table 6 and Figure 5B). The GKK1 group also showed higher HGH levels at 24 h (*p* < 0.001, 95% CI [0.01 to 0.05], Cohen’s *d* = 1.187) (Table 6 and Figure 5C).

GKK1 supplementation was associated with a lower sympathoadrenal response and better preservation of circulating dopamine after EIMD. At 3 h, the GKK1 group showed lower adrenaline (*p* = 0.022, 95% CI [−22.58 to −1.74], Cohen’s *d* = 0.683) and noradrenaline levels (*p* = 0.001, 95% CI [−76.99 to −23.01], Cohen’s *d* = 1.072) than the placebo group (Table 6 and Figure 5D,E). Because pre-EIMD dopamine concentrations differed significantly between the treatment groups (*p* = 0.008), the dopamine comparisons were reanalyzed using the baseline-adjusted ANCOVA described in Section 2.11, incorporating HC3 robust standard errors and Holm correction. Regarding dopamine, following ANCOVA adjustment for baseline discrepancies, the GKK1 group maintained a significantly higher adjusted mean concentration at 48 h (adjusted difference: +58.98 pg/mL, 95% CI [52.35 to 65.62], Holm adjusted *p* < 0.001) and a significantly greater percentage recovery trajectory (−14.38 ± 8.51% vs. −59.80 ± 4.31%, *p* < 0.001, 95% CI [41.47 to 49.37], Cohen’s *d* = 6.735) (Figure 5F). To systematically verify the robustness of the primary analytical models, secondary sensitivity analyses were conducted utilizing log-transformed baseline-adjusted models, evaluation of Cook’s distance, and model refitting subsequent to the exclusion of potentially influential observations. Crucially, these rigorous methodological evaluations yielded no material deviations in either the directionality or the statistical inferences of the principal biomarker findings.

### 3.8. Effects of GKK1 Supplementation on Urinary 3-Methylhistidine

At 24 h post-EIMD, urinary 3 methylhistidine concentrations were significantly lower in the GKK1 group than in the placebo group (91.35 ± 12.56 vs. 105.35 ± 11.24 nmol/mL, *p* < 0.001). These findings are consistent with, but do not establish, lower endogenous myofibrillar proteolysis because a strict meat-free dietary washout was not implemented. Furthermore, urinary creatinine concentrations were significantly higher in the GKK1 group (115.97 ± 26.12 vs. 80.48 ± 29.05 mg/dL, *p* < 0.001). Consistently, the 3-methylhistidine-to-urinary creatinine ratio was significantly lower in the GKK1 group (0.86 ± 0.40 vs. 1.46 ± 0.47, *p* < 0.001) (Table 7).

## 4. Discussion

The present randomized, double-blind, placebo-controlled trial demonstrates that four weeks of *L. plantarum* GKK1 supplementation significantly accelerates recovery from EIF and EIMD in healthy adult men. Our findings provide compelling evidence that the EIF phenotype is not merely a transient, localized reduction in force generation, but a complex, multi-systemic physiological challenge triggered by eccentric mechanical stress. This high-intensity plyometric model characteristically causes microstructural disruption of skeletal muscle fibers, leading to a cascade of transient declines in force production, explosive power, and anaerobic capacity symptoms that mirror established structural recovery profiles documented in the previous literature [25,28]. While prior investigations into the “gut–muscle axis” have predominantly viewed specific probiotic strains as isolated modulators of inflammatory signaling or endurance capacity [10,29], this study bridges the mechanistic gap between molecular catabolic suppression, systemic redox balance, and neuroendocrine homeostasis.

Transcending a compartmentalized view of these discrete biological markers, GKK1’s protective efficacy can be conceptualized as an upstream regulatory cascade that simultaneously preserves physical and neural capacity. The pathophysiological domino effect of EIMD initiates with severe mechanical shearing of the sarcolemma. This structural disruption triggers intracellular calcium overload, stimulating the calcium-dependent protease (calpain) system, which then upregulates the ubiquitin–proteasome pathway, ultimately leading to the degradation of the contractile apparatus and the rapid efflux of intramuscular enzymes into the systemic circulation [30]. In the present study, GKK1 supplementation effectively mitigated the downstream consequences of this proteolytic cascade, as evidenced by significantly lower absolute concentrations and relative percentage changes in serum CK and myoglobin, two definitive markers of sarcolemmal disruption and myofibrillar damage [31]. Crucially, this structural protection is corroborated by our urinary metabolic data; the GKK1 cohort displayed significantly lower urinary 3-MH levels and a diminished 3-MH/creatinine ratio post-EIMD. Because 3-methylhistidine is derived directly from actin and myosin degradation and cannot be reutilized for protein synthesis, its reduction provides suggestive physiological evidence that GKK1 limits myofibrillar proteolysis [32,33]. Crucially, this structural protection and the concurrent attenuation of systemic inflammation are biologically underpinned by the unique genomic characteristics of *L. plantarum* GKK1. The nitrate reductase region identified in GKK1 provides a plausible mechanistic basis for altered nitric oxide-related signaling; however, NO production and skeletal muscle perfusion were not directly assessed in the present trial. Concurrently, its distinct glucorhamnan-related gene clusters actively stimulate immunomodulatory cytokines, including interferon beta and interleukin 12, hypothesized to support a targeted attenuation of the secondary inflammatory cascade [20].

The structural integrity of myofibrils is inextricably linked to the optimization of cellular bioenergetics. During high-intensity supramaximal tasks such as the 30 s Wingate sprint, approximately 75% of the total energetic demand must be supplied by anaerobic metabolism [34]. This relies heavily on the rapid hydrolysis of intracellular adenosine triphosphate (ATP) and phosphocreatine (PCr) to drive maximal power output within a highly compressed timeframe [35]. Consequently, elevating intramuscular creatine reserves accelerates ATP resynthesis and sustains metabolic buffering capacity, thereby counteracting the acute energy deficit induced by high-intensity plyometric loading and mitigating subsequent exercise-induced structural damage [36]. Beyond the immediate availability of anaerobic substrates, this enhanced bioenergetic profile may also be driven by microbiota-mediated mitochondrial adaptations [37]. It is well established that the upregulation of these pathways, particularly through the robust expression of the mitochondrial biogenesis master regulator PGC 1α, markedly enhances mitochondrial respiratory function and amplifies overall cellular ATP production [38]. Evidence from murine models indicates that specific lactobacilli can counteract metabolic degradation by modulating the expression of the SIRT1/PGC 1α signaling axis, alongside downstream mitochondrial transcription factors like NRF1 and TFAM [39]. Therefore, building upon metabolic observations in endurance athletes supplemented with other L. plantarum strains [18], we postulate that GKK1 supplementation confers a dual metabolic advantage: potentially supporting the preservation of the immediate PCr pool through structural protection while potentially upregulating mitochondrial ATP synthetic capacity. This comprehensive biochemical optimization directly underpins the superior functional recoveries observed in the GKK1 group, translating into preserved relative mean and peak power, as well as expedited restoration of peak force and the pRFD during CMJ and IMTP assessments. While absolute mean differences between groups might appear numerically narrow due to high interindividual anatomical variation, analyzing the normalized percentage changes reveals a highly relevant clinical advantage. Rather than relying solely on mathematical *p* values, this preservation suggests a potential practical advantage. In applied sports settings, a reliable preservation in explosive capacity allows athletes to sustain repeated sprint ability, maintain higher mechanical output, and delay fatigue-induced biomechanical compensations during congested training schedules or the critical later stages of competition.

Critically, this peripheral inflammatory mitigation operates in tandem with an upstream, gut-mediated stabilization of the neuroendocrine and neuromodulatory axes, providing a synchronized defense against both central and peripheral fatigue pathways within a single regulatory continuum. Under standard EIMD conditions, elevated circulating inflammatory cytokines and accumulated lipid peroxides act as potent stimuli that hypersensitize unmyelinated Type IV and thinly myelinated Type III muscle afferents [40]. These peripheral afferents project to the central nervous system, delivering persistent inhibitory feedback that restricts alpha motor neuron excitability and downregulates motor unit recruitment. Conversely, by dampening the systemic inflammatory load [41], GKK1 is hypothesized to attenuate this peripheral inhibitory pathway, thereby sustaining a higher capacity for voluntary motor unit recruitment during mechanical exhaustion. This peripheral desensitization operates alongside the stabilization of systemic sympathetic tone. Notably, our neurological data demonstrate that GKK1 supplementation significantly mitigated the severe exercise-induced depletion of serum dopamine observed in the placebo group while attenuating acute adrenaline spikes. Because systemic catecholamines do not freely cross the blood–brain barrier, circulating dopamine and adrenaline reflect peripheral sympathetic nervous system activity and adrenal output rather than central neurotransmitter concentrations [42]. Therefore, this preservation indicates that GKK1 significantly decreases systemic stress and sympathetic overactivity, facilitating a more stable physiological environment for recovery. Furthermore, this protective neuro signaling loop is inextricably linked to the preservation of the systemic anabolic catabolic balance, effectively shifting the post-exercise systemic environment away from prolonged stress and toward accelerated structural remodeling [43]. Strenuous eccentric exercise typically disrupts systemic homeostasis, triggering prolonged hyperreactivity of the HPA axis that drives cortisol-mediated catabolism at the expense of testosterone-mediated tissue repair [44,45]. However, in the present study, GKK1 significantly preserved absolute serum testosterone concentrations and facilitated an expedited normalization of HGH kinetics, both of which are critical anabolic drivers essential for stimulating muscle protein synthesis, optimizing glycogen resynthesis, and coordinating structural tissue remodeling [46,47]. From a mechanistic perspective, this reciprocal neuroendocrine regulation is hypothesized to be mediated by the enhanced microbial synthesis of short-chain fatty acids, such as butyrate, which can cross the blood–brain barrier or engage vagal pathways to effectively modulate HPA axis sensitivity and stress responsiveness [10,18]. We hypothesize that GKK1-mediated enrichment of the intestinal microenvironment optimizes the systemic kinetics of these neuroactive metabolites, thereby suppressing excess glucocorticoid secretion and shielding circulating androgens from stress-induced suppression. Consequently, this comprehensive neuroendocrine optimization preserves the physiological timeline required for muscular remodeling and homeostatic adaptation. While these clinical observations provide a sophisticated neurophysiological framework, future investigations incorporating microbiome sequencing, fecal metabolomics, and direct neuromuscular assessments are warranted to definitively elucidate these gut–brain and gut–muscle mechanistic pathways.

### Limitations of the Study

The clinical utility of *L. plantarum* GKK1 is supported by its favorable safety and tolerability profile, alongside rigorous quality control. Four weeks of high-dose daily supplementation did not adversely alter clinical biomarkers of liver function, renal function, blood glucose, or lipid profiles, confirming its suitability for regular dietary inclusion in sedentary populations. Furthermore, while all participants performed the standardized 90° knee flexion, actual mechanical loading and deceleration work during the EIMD protocol were not directly quantified, representing an inherent limitation in standardizing true muscular workload. Nevertheless, several methodological limitations must be explicitly acknowledged when interpreting these findings. First, to intentionally control for biological and hormonal variance, the trial was conducted exclusively in healthy young adult males. Consequently, further research is required to determine whether these recovery benefits generalize to female athletes, older adults experiencing sarcopenia, highly trained elite competitors, or clinical populations. Second, the physiological benefits observed in this trial must be interpreted as strictly specific to the tested GKK1 formulation. Given the permissive regulation of dietary supplements globally and widespread commercial issues regarding batch viability and chemical contamination, our findings cannot be generalized to other commercially available *L. plantarum* products. Third, while the physiological and biochemical data strongly point toward a gut-mediated mechanism, our protocol did not perform direct sequencing of the gut microbiota or fecal and systemic SCFA quantification. Therefore, the proposed mechanisms regarding short-chain fatty acid-mediated recovery remain speculative theoretical hypotheses, limiting our ability to definitively trace specific bacterial taxa shifts. Fourth, although dietary protein and meat intake were strictly monitored and balanced between groups, a complete meat-free washout was not enforced. This lack of restriction may introduce minor confounding variations in urinary 3-methylhistidine derived from dietary sources. Fifth, the intervention framework was restricted to a four-week timeline, leaving the long-term adaptations and potential ceiling effects of chronic GKK1 intake unexamined. Sixth, while the placebo and probiotic capsules were visually and physically identical, a formal statistical evaluation of blinding success via end-of-trial participant guessing was not conducted. Finally, post-exercise blood biomarker concentrations were not mathematically adjusted for plasma volume changes. However, the short duration of the plyometric protocol and the standardized hydration controls likely minimized severe hemoconcentration artifacts. To resolve these academic gaps, subsequent research designs should implement multi-dose–response profiling, incorporate diverse training demographics of both sexes, and utilize high-throughput metagenomic and metabolomic sequencing.

In conclusion, the present study suggests that *L. plantarum* GKK1 supplementation may be a useful nutritional strategy for supporting recovery from EIF and EIMD. GKK1 was associated with better preservation of neuromuscular performance, lower markers of muscle damage and oxidative/inflammatory stress, reduced urinary indices of myofibrillar proteolysis, and a more favorable post-exercise endocrine profile. Because direct gut microbiota and mechanistic neuroendocrine measurements were not performed, the proposed gut–muscle–neuro interpretation should be considered hypothesis-generating and should be confirmed in future mechanistic trials.

## 5. Conclusions

In conclusion, this randomized and placebo-controlled trial suggests that 28 days of *L. plantarum* GKK1 supplementation may support selected indices of post-EIMD recovery in sedentary young men. Specifically, while GKK1 cannot physically prevent the initial mechanical microtrauma caused by strenuous loading, it is associated with the attenuation of the secondary inflammatory cascade and accelerates functional recovery kinetics, evidenced by preserved countermovement jump, isometric strength, and anaerobic power. This functional preservation is systematically mirrored by marked reductions in circulating biomarkers of muscle damage, systemic inflammation, lipid peroxidation, and urinary excretion of 3-methylhistidine, while concurrently fostering a favorable anabolic endocrine environment. Crucially, these extensive physiological benefits are achieved with excellent clinical safety. These findings warrant confirmation in trained athletic populations before sport-specific recommendations or optimal preloading phases can be established. Although we hypothesize that these broad systemic benefits are mediated by gut microbiota modulation and specific upstream pathways, this remains a speculative mechanism. To fully substantiate these findings, future research must utilize skeletal muscle biopsies to directly assess intramuscular inflammatory signaling alongside advanced metagenomic and metabolomic sequencing to confirm the exact regulatory pathways.

## Figures and Tables

**Table 1:** Participant basic information.

Characteristics	Placebo	GKK1
Age (Year)	22.3 ± 1.9	22.3 ± 2.2
Height (cm)	177.2 ± 6.7	173.9 ± 4.7
Weight (kg)	73.9 ± 7.8	70.0 ± 7.5

Data are presented as mean ± SD.

**Table 2:** Effects of GKK1 Supplementation on Grip Strength.

Characteristics	Before		After	
	Placebo	GKK1	Placebo	GKK1
Left (kg)	43.9 ± 5.3	42.2 ± 5.8	42.9 ± 6.0	44.5 ± 5.5
Right (kg)	44.8 ± 6.4	43.3 ± 4.1	44.0 ± 7.1	44.8 ± 4.4

Data are presented as mean ± SD.

**Table 3:** Effects of GKK1 Supplementation on Countermovement Jump Recovery.

Variable	Group	Pre-EIMD	Post 3 h	Post 24 h	Post 48 h	Group *p*	Time *p*	G × T *p*
RFD (N/kg/s)	Placebo	9.95 ± 0.97	8.63 ± 1.21	7.23 ± 0.98 ^a^	7.75 ± 0.84 ^a^	0.032	<0.001	0.024
	GKK1	10.00 ± 0.85	8.92 ± 1.17	7.92 ± 1.19 ^b^	8.73 ± 1.06 ^b^			
Relative peak force (N/kg)	Placebo	15.45 ± 1.45	14.55 ± 1.48	13.24 ± 1.18	14.12 ± 1.39 ^a^	0.260	<0.001	<0.001
	GKK1	15.48 ± 1.36	14.84 ± 1.45	14.00 ± 1.38	14.94 ± 1.40 ^b^			
Jump height (m)	Placebo	0.32 ± 0.03	0.32 ± 0.03	0.30 ± 0.03	0.31 ± 0.03	0.523	<0.001	0.006
	GKK1	0.32 ± 0.04	0.32 ± 0.03	0.31 ± 0.03	0.32 ± 0.03			

Data are presented as mean ± SD. Different superscript letters indicate significant differences between groups at the same time point (*p* < 0.05).

**Table 4:** Effects of GKK1 Supplementation on Isometric Mid-Thigh Pull Performance.

Variable	Group	Pre-EIMD	Post 3 h	Post 24 h	Post 48 h	Group *p*	Time *p*	G × T *p*
Relative peak force (N/kg)	Placebo	15.39 ± 1.29	14.14 ± 1.23	13.00 ± 1.16 ^a^	13.54 ± 1.29 ^a^	0.054	<0.001	<0.001
	GKK1	15.52 ± 1.36	14.71 ± 1.38	13.87 ± 1.27 ^b^	14.79 ± 1.34 ^b^			
pRFD (N/s)	Placebo	9228 ± 926	8389 ± 798	7413 ± 796	8159 ± 813 ^a^	0.306	<0.001	<0.001
	GKK1	9179 ± 690	8493 ± 731	7779 ± 739	8633 ± 710 ^b^			

Data are presented as mean ± SD. Different superscript letters indicate significant differences between groups at the same time point (*p* < 0.05).

**Table 5:** Effects of GKK1 Supplementation on Wingate Anaerobic Power.

Variable	Group	Pre-EIMD	Post 3 h	Post 24 h	Post 48 h	Group	Time	G × T
Relative mean power (W/kg)	Placebo	6.44 ± 0.41	5.78 ± 0.50 ^a^	5.29 ± 0.46 ^a^	5.78 ± 0.44 ^a^	0.003	<0.001	<0.001
	GKK1	6.63 ± 0.50	6.20 ± 0.57 ^b^	5.81 ± 0.49 ^b^	6.33 ± 0.50 ^b^			
Relative peak power (W/kg)	Placebo	9.42 ± 0.97	8.69 ± 0.91	7.84 ± 0.73	8.42 ± 0.77 ^a^	0.373	<0.001	0.060
	GKK1	9.47 ± 0.82	8.85 ± 0.82	8.29 ± 0.82	8.93 ± 0.76 ^b^			
Fatigue index (%)	Placebo	48.5 ± 2.9	54.9 ± 3.7	60.7 ± 3.2 ^b^	56.0 ± 2.6 ^b^	0.476	<0.001	0.140
	GKK1	48.6 ± 2.9	53.1 ± 3.1	57.6 ± 2.9 ^a^	52.8 ± 2.9 ^a^			

Data are presented as mean ± SD. Different superscript letters indicate significant differences between groups at the same time point (*p* < 0.05).

**Table 6:** Effects of GKK1 Supplementation on Muscle Damage, Inflammatory Neuroendocrine and Metabolic Profiling Biomarkers.

Blood Parameters		Pre- EIMD	Post-EIMD 3 h	Post-EIMD 24 h	Post-EIMD 48 h	ANOVA *p*-Value		
						Group	Time	G× T
CK (U/L)	Placebo	152 ± 16	188 ± 19	423 ± 48 ^b^	318 ± 48 ^b^	<0.001	<0.001	<0.001
	GKK1	149 ± 22	177 ± 23	324 ± 48 ^a^	214 ± 27 ^a^			
Myoglobin (ng/mL)	Placebo	21.0 ± 4.0	98.4 ± 11.2 ^b^	29.5 ± 4.0 ^b^	23.2 ± 3.6	<0.001	<0.001	<0.001
	GKK1	21.9 ± 2.3	72.9 ± 7.3 ^a^	27.3 ± 2.7 ^a^	23.4 ± 2.3			
hs-CRP (mg/dL)	Placebo	0.08 ± 0.01	0.10 ± 0.01	0.25 ± 0.03 ^b^	0.18 ± 0.02 ^b^	<0.001	<0.001	<0.001
	GKK1	0.09 ± 0.01	0.10 ± 0.01	0.18 ± 0.02 ^a^	0.12 ± 0.02 ^a^			
Testosterone (ng/mL)	Placebo	5.43 ± 0.44	3.91 ± 0.37 ^a^	4.46 ± 0.39 ^a^	4.84 ± 0.40	0.060	<0.001	<0.001
	GKK1	5.36 ± 0.52	4.39 ± 0.57 ^b^	4.76 ± 0.45 ^b^	5.09 ± 0.49			
TBARS (μM)	Placebo	7.08 ± 0.81	9.47 ± 0.81 ^b^	15.61 ± 1.04 ^b^	12.86 ± 1.18 ^b^	<0.001	<0.001	<0.001
	GKK1	7.08 ± 0.59	8.85 ± 0.65 ^a^	13.36 ± 0.90 ^a^	10.13 ± 0.85 ^a^			
Cortisol (μg/dL)	Placebo	13.10 ± 4.35	9.62 ± 3.06	9.67 ± 3.12	6.90 ± 2.41 ^b^	0.963	<0.001	0.008
	GKK1	13.11 ± 5.13	12.05 ± 5.56	8.59 ± 2.91	5.39 ± 2.02 ^a^			
HGH (ng/mL)	Placebo	0.07 ± 0.03	0.12 ± 0.03 ^b^	0.10 ± 0.03 ^a^	0.13 ± 0.04 ^a^	0.006	<0.001	<0.001
	GKK1	0.06 ± 0.02	0.10 ± 0.02 ^a^	0.13 ± 0.02 ^b^	0.24 ± 0.12 ^b^			
Adrenaline (pg/mL)	Placebo	11.17 ± 1.21	47.39 ± 18.38 ^b^	36.30 ± 12.35	11.54 ± 1.11	0.455	<0.001	0.007
	GKK1	10.84 ± 0.86	35.23 ± 17.22 ^a^	43.03 ± 20.67	11.23 ± 0.96			
Noradrenaline (pg/mL)	Placebo	114 ± 29	326 ± 49 ^b^	270 ± 46 ^b^	217 ± 42 ^b^	<0.001	<0.001	<0.001
	GKK1	146 ± 30	277 ± 43 ^a^	185 ± 39 ^a^	147 ± 30 ^a^			
Dopamine (pg/mL)	Placebo	130 ± 11 ^b^	72 ± 6	59 ± 5 ^a^	52 ± 6 ^a^	<0.001	<0.001	<0.001
	GKK1	122 ± 9 ^a^	73 ± 7	74 ± 7 ^b^	105 ± 16 ^b^			

Data are presented as mean ± SD. Different superscript letters indicate significant differences between groups at the same time point (*p* < 0.05).

**Table 7:** Effects of GKK1 Supplementation on Urinary 3-Methylhistidine.

Urine	Placebo	GKK1
3-methylhistidine (nmol/mL)	105.35 ± 11.24 ^b^	91.35 ± 12.56 ^a,^
Urinary creatinine (mg/dL)	80.48 ± 29.05 ^a^	115.97 ± 26.12 ^b^
3-methylhistidine/urinary creatinine (ratio)	1.46 ± 0.47 ^b^	0.86 ± 0.40 ^a^

Data are presented as mean ± SD. Different superscript letters indicate significant differences between groups at the same time point (*p* < 0.05).

## Data Availability

The data presented in this study are available within the article.

## Supplementary Materials

The following supporting information can be downloaded at: https://www.mdpi.com/article/10.3390/nu18172782/s1, Table S1: Subject’s dietary intake before and after the 4-week GKK1 intervention; Table S2. Subject’s body composition before and after the 4-week GKK1 intervention; Table S3. Subject’s blood biochemical parameters before and after the 4-week GKK1 intervention.
