# Supplementary material for: Effect of Lactiplantibacillus plantarum GKK1 Supplementation on Exercise-Induced Fatigue, Muscle Damage, and Recovery in Healthy Men: A Randomized, Double-Blind, Placebo-Controlled Trial
Source: Nutrients. 2026 Aug 25;18(17):2782. doi: 10.3390/nu18172782 (PMC13567057; doi:10.3390/nu18172782)
Supplement: Supplementary file 1 [file nutrients-18-02782-s001.zip › Table S1.pdf]

Table S1. Subject's dietary intake before and after the 4-week GKK1 intervention.

Characteristics	Before		After	
	Placebo	GKK1	Placebo	GKK1
Carbohydrate (g)	243±25 [a]	233±27 [a]	243±27 [a]	235±38 [a]
Protein (g)	120±20 [a]	114±16 [a]	117±16 [a]	113±11 [a]
Fat (g)	99±7 [a]	99±11 [a]	99±9 [a]	97±8 [a]
Total calorie (kcal)	2345±192 [a]	2278±205 [a]	2334±203 [a]	2270±211 [a]

Data are presented as mean ± SD.
