# Supplementary material for: Effect of Lactiplantibacillus plantarum GKK1 Supplementation on Exercise-Induced Fatigue, Muscle Damage, and Recovery in Healthy Men: A Randomized, Double-Blind, Placebo-Controlled Trial
Source: Nutrients. 2026 Aug 25;18(17):2782. doi: 10.3390/nu18172782 (PMC13567057; doi:10.3390/nu18172782)
Supplement: Supplementary file 1 [file nutrients-18-02782-s001.zip › Table S2.pdf]

Table S2. Subject's body composition before and after the 4-week GKK1 intervention.

Characteristics	Before		After	
	Placebo	GKK1	Placebo	GKK1
Body Weight (kg)	73.9±7.8 [a]	70.0±7.5 [a]	73.9±7.8 [a]	70.0±7.5 [a]
BMI (kg/m^2)	23.6±2.3 [a]	23.2±2.4 [a]	23.6±2.3 [a]	23.2±2.4 [a]
Muscle mass (kg)	34.5±3.9 [a]	32.6±3.4 [a]	34.5±3.9 [a]	32.6±3.4 [a]
Fat mass (%)	17.7±6.8 [a]	16.5±5.8 [a]	17.7±6.8 [a]	16.5±5.8 [a]

Data are presented as mean ± SD. The same superscript letters (a) indicate no significant difference between groups at $p > 0.05$. BMI, body mass index
