# Supplementary material for: Effect of Lactiplantibacillus plantarum GKK1 Supplementation on Exercise-Induced Fatigue, Muscle Damage, and Recovery in Healthy Men: A Randomized, Double-Blind, Placebo-Controlled Trial
Source: Nutrients. 2026 Aug 25;18(17):2782. doi: 10.3390/nu18172782 (PMC13567057; doi:10.3390/nu18172782)
Supplement: Supplementary file 1 [file nutrients-18-02782-s001.zip › Table S3.pdf]

Table S3. Subject's blood biochemical parameters before and after the 4-week GKK1 intervention.

Characteristics	Before		After	
	Placebo	GKK1	Placebo	GKK1
AST (U/L)	25±6 [a]	23±5 [a]	25±5 [a]	24±6 [a]
ALT (U/L)	26±7 [a]	24±6 [a]	26±6 [a]	23±5 [a]
TC (mg/dL)	181±13 [a]	186±11 [a]	182±15 [a]	186±11 [a]
TG (mg/dL)	96±17 [a]	100±17 [a]	98±17 [a]	100±16 [a]
HDL (mg/dL)	63.4±10.8 [a]	59.7±9.3 [a]	62.4±10.5 [a]	58.3±9.0 [a]
LDL (mg/dL)	101.6±16.4 [a]	109.1±11.9 [a]	104.1±13.9 [a]	110.2±9.1 [a]
BUN (mg/dL)	16.4±4.6 [a]	16.2±5.0 [a]	16.5±4.4 [a]	16.5±3.3 [a]
CREA (mg/dL)	1.19±0.07 [a]	1.23±0.05 [a]	1.18±0.08 [a]	1.20±0.12 [a]
UA (mg/dL)	6.7±0.8 [a]	6.7±0.8 [a]	6.7±0.8 [a]	6.7±0.6 [a]
Glucose (mg/dL)	80±11 [a]	81±11 [a]	79±11 [a]	81±12 [a]

Data are presented as mean ± SD. The same superscript letters (a) indicate no significant difference between groups at $p > 0.05$. AST, aspartate aminotransferase; ALT, alanine transaminase; TC, total cholesterol; TG, triacylglycerol; HDL-C, high-density lipid cholesterol; LDL-C, low-density lipid cholesterol; BUN, blood urea nitrogen; CREA, creatinine; UA, uric acid.
